# Exploring the molecular landscape of lymphocyte activation gene-3: A literature review

**DOI:** 10.1097/MD.0000000000039622

**Published:** 2024-09-27

**Authors:** Jiaqi Nie, Xue Qin, Xiang Tao, Jin Huang

**Affiliations:** a Clinical Laboratory Center, The First Affiliated Hospital of Guangxi Medical University, Nanning, China.

**Keywords:** IC, immune homeostasis, LAG-3, TME

## Abstract

Molecular structure and cellular distribution of lymphocyte activation gene-3 (LAG-3) have been studied extensively since 1990. However, several unresolved questions remain. It is well-established that LAG-3 plays a significant role in maintaining immune homeostasis. The presence of deficiencies in LAG-3 has been observed to be linked with autoimmune disorders, whereas the excessive expression of LAG-3 within the tumor microenvironment hinders immune responses, particularly those mediated by lymphocytes, thereby facilitating immune evasion. Consequently, investigations into these 2 aspects have become a prominent focus in both fundamental and clinical research. The objective of this review is to examine the functions and molecular characteristics of LAG-3, as well as its current clinical applications in the context of tumor immune escape and autoimmune disease. The ultimate aim is to explore and propose novel immune therapy approach.

## 1. Introduction

Familiar to programmed death-1 (PD-1), lymphocyte activation gene-3 (LAG-3) plays a crucial role in maintaining immune tolerance as a novel immune checkpoint (IC). Specific therapies have been developed and applied accordingly. However, the role of LAG-3 in signaling transmission and its interaction with other ICs remains a topic of research controversy and uncertainty, which is of great significance in understanding the development of diseases.

In terms of autoimmunity, an imbalance in immune homeostasis, where T cells are overactivated without any limitations, is the underlying cause of most autoimmune disorders. This overactivation leads to damage in normal tissues and organs. To avoid this kind of damage, T cells are regulated by ICs such as LAG-3, which helps maintain immune homeostasis, specifically in the signal transmitting pathway.^[1–4]^ Within the context of tumor development, it has been observed that LAG-3 within the lysosome of T cells, undergoes translocation to the cell surface through the action of lipid raft. This translocation is facilitated by the activation of protein kinase C (PKC) signaling.^[5]^ This allows LAG-3 to transmit suppressive signals to T cells when it binds to its ligands, such as fibrinogen-like protein 1 (FGL1) and galectin-3 (Gal-3). This leads to T-cell exhaustion and tumor immune evasion.^[6]^

In this study, we comprehensively examined the characteristics of the molecule LAG-3, including its expression, specific function, relevance to disease, and potential application as a therapeutic target. To fully leverage the benefits of immune therapies, it is imperative to gain a comprehensive understanding of the role of LAG-3 in the immune system.

## 2. Structure and function

The lag-3 gene is present in humans on chromosome 12, while in mice it is located on chromosome 6. This gene encodes a type I membrane protein with a weight of 70 kDa and consists of 498 amino acids.^[7]^ LAG-3 is primarily found within late endosomes and lysosomes, where it is stored and degraded. The aforementioned process plays a crucial role in the regulation of LAG-3 levels in unstimulated T cells, thus playing a significant role in maintaining immune homeostasis.^[8]^ After the activation of T cells, LAG-3, which is stored within the lysosome, can be released, and its cytoplasmic domain can be recognized by a receptor located on the cell membrane. This recognition process is accompanied by conformational alterations that are facilitated by PKC. As a result, LAG-3 is able to localize and be expressed on the cell surface. The precise characteristics of the recognition receptor and its relation to PKC remain unclear.^[5]^

In terms of its structural composition, LAG-3 can be categorized into 3 distinct regions: extracellular, transmembrane, and intracellular. There are 4 domains in the extracellular region, D1 to D4. As an immunoglobulin variable-like region, D1 primarily binds to MHCII molecules. Although its affinity is higher than that of CD4,^[9]^ it lacks the competitive inhibition capability to suppress the signal transmission ability of CD4.^[10]^ In the interim, D1 and D2 serve as the main junctions for fibrinogen-like protein 1 (FGL-1). Their role in transmitting the mediating signals induced by FGL-1 can overlap.^[11]^ Regarding D4, there exists a connecting peptide between this domain and the transmembrane region. This connecting peptide can be cleaved by ADAM17, a process that is mediated by ADAM10 under T-cell receptor signaling. As a result of this cleavage, the extracellular region transforms into sLAG-3.^[12,13]^ On one hand, it has been demonstrated that the presence of sLAG-3 can augment the proliferation and immune functionality of CD8+ T and Th1 cells.^[14]^ On the other hand, the level of sLAG-3 in peripheral blood can serve as a prognostic biomarker for patients undergoing treatment, although its significance varies depending on the type of cancer.^[15–18]^ The elucidation of the physical and pathological significance of sLAG-3 requires further investigation.

The intracellular region consists of 3 motifs, namely FXXL, KIEEKE, and EPrepeats. Research studies have demonstrated that this particular region plays a crucial role in the transmission of LAG-3 signaling. Without this region, LAG-3 loses its function entirely. However, the specific impact of motifs on signal transmission remains unclear. The FXXL motif, as the prevailing perspective suggests, implies that IL-12 has the ability to bind to it, consequently leading to the inhibition of IL-12 secretion. The presence of KIEELE is not essential for the transmission of LAG-3 signaling. Additionally, EPrepeats may be involved in the cascade reaction between LAG-3 and CD3, CD4, and CD8. After LAG-3 relevant proteins were connected with EPrepeats, the CD3/TCR signaling pathway can be effectively inhibited when there is an erroneous activation from the coreceptors such as CD4. LAG-3 continues to exhibit activity even in the absence of this motif, indicating that its presence for signal transmission is also dispensable.^[6,9]^ To summarize, there are still unresolved controversies and gaps in our understanding of the molecular mechanisms underlying the synergistic and individual effects of motifs in signal transmission.

## 3. Cell expression

In relation to T cells, LAG-3 primarily plays a role in regulating the proliferation of naive T cells through metabolic processes, thereby maintaining a dynamic equilibrium. For naive T cells, researchers have discovered that LAG-3 plays a significant role in mitochondrial stasis. However, it is not naturally expressed on the cell surface.^[19]^ This results in a reduced metabolic efficiency, thereby slowing down the proliferative rate of naïve T cells. Further research has shown that naive CD4+ T cells express less LAG-3 on their surface compared to activated or exhausted CD4+ T cells. In a LAG-3 deficient situation, naive CD4+ T cells exhibit a better response to stimulation, including improved metabolism and proliferation ability.^[20]^ Under conditions of sustained antigen stimulation and in the presence of the microtubule organizing center, CD4+ and CD8+ T cells upregulated their expression of LAG-3 on their cell membrane. This upregulation ultimately results in impaired proliferation and diminished immune response efficacy. Consequently, T cells become functionally exhausted and lose their immune ability. This phenomenon is particularly evident in tumor infiltrating T cells, which exhibit this characteristic as a hallmark feature.^[6]^ In a situation where LAG-3 is deficient, the activation of signal transducer and activator of transcription occurs, leading to an enhancement in the glycolytic and immune function of naïve T cells. This activation also results in the downregulation of the metabolic regulation of CD4+ T cells in response to IL-7.^[20]^ In the context of metabolic processes, it has been discovered through research that LAG-3 plays a key role in the memory effect by maintaining oxidative phosphorylation in CD8+ T cells.^[21]^ Notably, the expression of LAG-3 on T cells can be induced by various cytokines, including TNF, IL-7, and IL-2.^[22]^

Regarding regulatory T cells (Tregs), there is still a positive correlation between the level of LAG-3 and stimulation. This increase in LAG-3 expression leads to the suppression of proliferation and immune function, ultimately contributing to the maintenance of immune homeostasis. Among the various subsets of Tregs, Tr1 cells are known to exhibit high expression of LAG-3. Co-expression of CD49b and LAG-3 has been identified as a potential recognition feature for Tr1 cells.^[23,24]^ In the context of feature function, the most significant question is determining the mechanism by which LAG-3 interferes with the suppressive effect of Tregs. Two in vivo experiments have yielded contrasting results regarding the impact of Tregs on T cells. Therefore, further investigation is required to elucidate the mechanism of LAG-3’s regulatory role in Tregs.^[25,26]^

Regarding dendritic cells (DCs), LAG-3 is primarily found in plasmacytoid dendritic cells and human monocyte-derived dendritic cells. In one aspect, LAG-3 plays a role in regulating immune homeostasis and promoting the secretion of TNF-α and IL-27 by mature DCs.^[27]^ In another aspect, the absence of LAG-3 causes a metabolic shift in antigen presenting cells (APCs) from oxidative phosphorylation to glycolysis. This leads to an increase in TNF-α secretion and a decrease in IL-10 secretion.^[21]^

Natural killer T cells and their subsets also express LAG-3, but there is no correlation between the expression level of LAG-3 and their cytotoxic function.^[28]^ LAG-3 is not the sole upregulated IC following stimulation of invariant natural killer T cells, PD-1 is also upregulated. These 2 ICs work synergistically to prevent the occurrence of aberrant autoimmune damage.^[29]^ In conclusion, as a regulator of immune homeostasis, LAG-3 possesses the capacity to collaborate with other ICs in order to modulate the production of cytokines. This ability allows LAG-3 to actively participate in the metabolic processes of various lymphocytes, ultimately leading to a balanced state of activation and suppression within the cellular context. Further investigation and clarification are required to understand the detailed mechanism by which LAG-3 influences non-T cells, including B and natural killer cells.

It is worth noting that LAG-3 is expressed on neurons and microglia cell surface and has the ability to specifically bind to α-synuclein (α-syn).^[30,31]^

## 4. Ligands

### 4.1. MHCII

As previously stated, LAG-3 exhibits a similar structure to CD4 and interacts with MHCII through its D1 motif. Notably, LAG-3 demonstrates a higher affinity for MHCII than CD4.^[7,32]^ However, once the intracellular tail of LAG-3 is cleaved, T cells are completely liberated from the constraints imposed by LAG-3. This indicates that competitive inhibition is not the primary mechanism underlying the relationship between LAG-3 and CD4.^[9,33]^ Despite the fact that the interaction between MHCII and LAG-3 does not elicit significant immune responses in lymphocytes, it is noteworthy that peptide MHCII (pMHCII) serves as the actual functional ligand.

MHCII transactivator (CIITA) is a cDNA molecule derived from cells that have acquired the ability to bind to LAG-3. Cell not only exhibit a low level of MHCII expression, also an inability to bind to the extra cellular zone of LAG-3 when CIITA is silenced. Further studies have revealed that the CIITA coding molecules Li and H2-DM play a crucial role in facilitating the formation of stable pMHCII, making them the primary accessory molecules.^[34,35]^ Additionally, LAG-3 possesses the capability to discern the structure of pMHCII and selectively bind to it. Therefore, this process plays a crucial role in modulating the classic antigen presenting pathway. As anticipated, the activation of CD4+ and CD8+ T cells, which possess the ability to recognize pMHCII are inhibited by LAG-3 when APCs secrete a substantial quantity of pMHCII.^[34]^ This procedure functions similarly to the immune evasion mechanism observed in tumor tissue.^[36,37]^ Further investigation is needed to understand the intracellular signal transmitting mechanism after their combination.

### 4.2. Fibrinogen-like protein 1

FGL-1 is widely recognized as a significant ligand of LAG-3, exhibiting a high degree of affinity between them. Additionally, FGL-1 plays a crucial role in the induction of LAG-3’s immunosuppressive effect. FGL-1 is primarily secreted by hepatocytes and plays a key role in the glycolytic pathway and liver cell regeneration under normal physiological conditions. It has been observed that IL-6 can enhance this process.^[11]^ Solid tumors, including lung, melanoma, colorectal, and breast tumors, secrete a significant amount of FGL-1. This secretion has been associated with poor prognosis and resistance to immunotherapy.^[38,39]^ The effects resulting from the interaction between LAG-3 and FGL-1 are distinct and not redundant to MHCII.^[7,9,33]^

For its function, the independent utilization of FGL-1 is not possible without the presence of LAG-3. FGL-1 has been shown to enhance the expression of LAG-3, thereby improving its immunosuppressive function. Specifically, it restricts the proliferation and secretion capabilities of CD8+ T cells. However, this phenomenon is not solely attributed to the direct impact of FGL-1/LAG-3. Instead, it is caused by indirect interference with the antigen presenting pathway of APCs. The specific mechanism underlying this process is still not fully understood. Further in vivo experimentation demonstrated that the injection of monoclonal antibodies (McAbs) targeting LAG-3, PD-1, and FGL-1 led to a modest inhibition of tumor growth. However, when anti-PD-1 was combined with either LAG-3 or FGL-1, a significant antitumor effect was observed. This suggests that FGL-1 plays a prominent role in tumor growth and invasion.^[11]^

Recent studies present varying perspectives. Initially, researchers have discovered that FGL-1 exhibits the ability to bind to T cells that lack LAG-3, indicating that LAG-3 is not the sole receptor binding to FGL-1. In addition, an in vitro experiment revealed that FGL-1 does not play a role in the suppressive function of LAG-3 on CD8+ T cells. Additionally, the presence of additional FGL-1 in the supportive environment does not enhance the immunosuppressive function of LAG-3.^[10]^ Another study demonstrated that tumor growth in FGL-1KO mice was effectively controlled, resulting in a favorable prognosis. However, this phenomenon was not observed in an in vitro setting.^[40]^ Combined with both traditional and contemporary perspectives, it is highly likely that FGL-1 functions by mediating the antigen presenting function of APCs. However, it does not directly impact tumor growth through the LAG-3 pathway on CD8+ T cells. This aspect also presents an opportunity for future research.

### 4.3. Galectin-3

Gal-3 has been identified as a noteworthy ligand for LAG-3 in the tumor microenvironment (TME). The combined effect of Gal-3 and LAG-3 has been found to be distinct from the combined effect of LAG-3 and MHCII, as reported in a study.^[41]^ Tumor-specific CD8+ T cells and stromal cells within the TME constitute the primary origin of Gal-3. After binding to LAG-3, Gal-3 interacts directly with CD8+ T cells and suppresses their immune function. Simultaneously, it downregulates the antigen presenting capability of APCs, thereby inhibiting the activation of CD8+ T cells. These 2 mechanisms interfere with the tumor-specific immune response, as evidenced by previous studies.^[42–44]^ Regarding the mechanism, an in vitro experiment revealed that Gal-3 has the ability to induce TCR glycosylation and cause irreversible changes to the T-cell genome. However, these phenomena were not observed in vivo.^[41]^

### 4.4. Liver sinusoidal endothelial cell lectin

LESCtin, like other inflammatory factors, originates from hepatocytes. Presenting in thymic dendritic cells of peripheral blood facilitates participation in anti-infection and cell adhesion processes. They also play a crucial role in the transmission and clearance of glycoproteins.^[45,46]^ Early research has indicated that the binding of LESCtin to LAG-3 facilitates tumor escape by suppressing the function of T cells in melanoma.^[46]^ Recent research has revealed that lymphocytes in the TME secrete LESCtin at extremely low levels, thereby indicating minimal impact of this ligand.^[47]^ The precise role of this factor in different types of cancer remains uncertain.

### 4.5. α-Synuclein

In the context of the neuronal system, both microglia and neurons demonstrate a significant level of LAG-3 expression. Furthermore, it has been determined that α-syn serves as the principal ligand for LAG-3 in this specific scenario.^[48]^ Their combination results in neuronal system damage, particularly through the facilitation of α-syn transmission in neurons. The sequencing experiment revealed that amyloid β precursor-like protein 1 has the potential to improve this process through its interaction with LAG-3. However, the exact signaling pathway responsible for this effect is still unknown. A-synuclein preformed fibrils, primarily elicits neuronal transformation and plays a significant role in the pathogenesis of Parkinson disease. The inhibition of this process can be achieved through the use of anti-LAG-3 antibodies, which effectively hinder the interaction between LAG-3 and amyloid β precursor-like protein 1.^[49]^ Apparently, LAG-3 not only plays a crucial role in the context of cancer, but it also has significant implications in the neuron system, necessitating further investigation.

**Figure 1.**
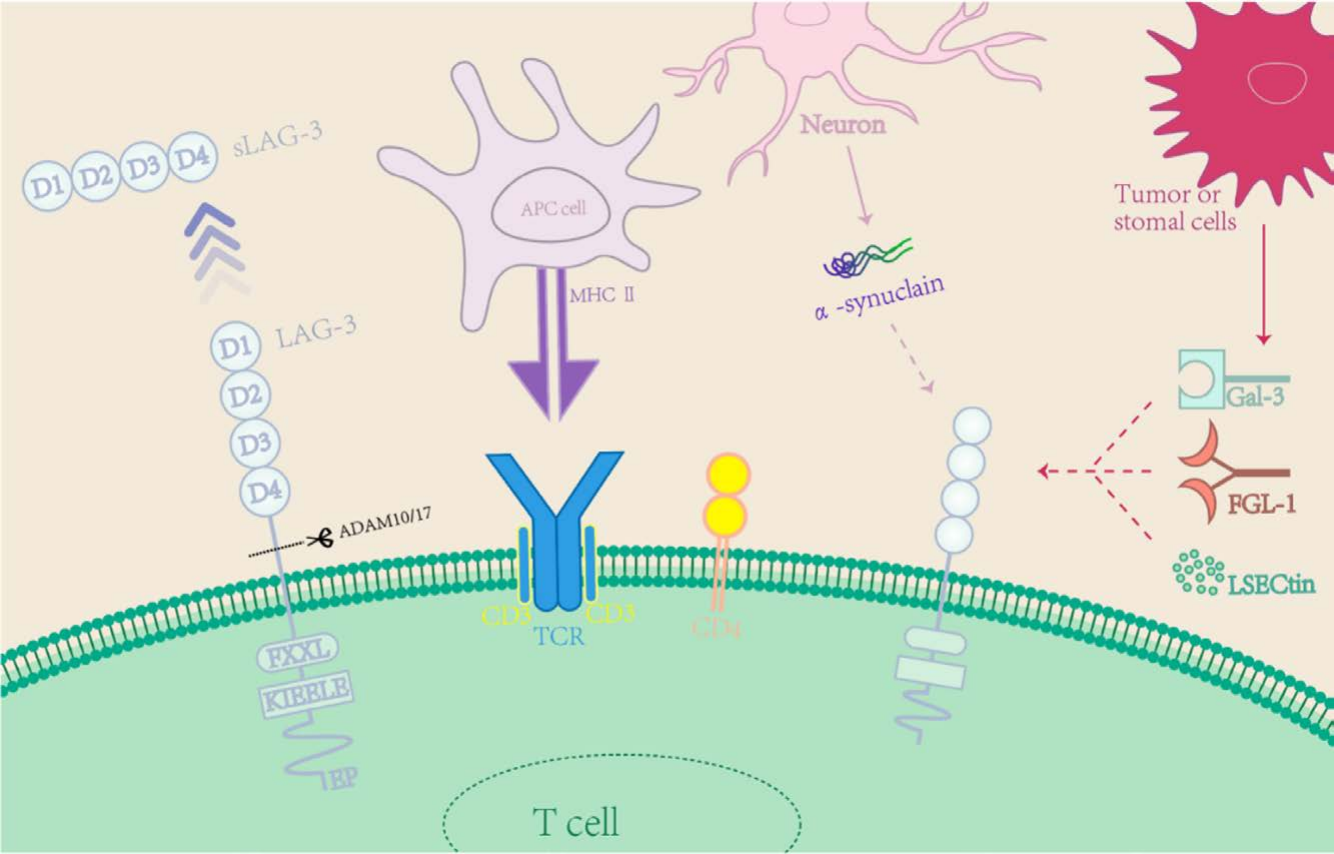
Structure and ligands of LAG3. MHC-II, galectin-3, LSECtin, FGL1, and α-synuclein were considered as ligands of LAG3. The origin cells of different ligands.

And finally, the structure and the relationship between LAG-3 and its ligands were shown in Figure 1.

## 5. Diseases and clinical application

### 5.1. Autoimmunity

As previously stated, LAG-3, functioning as a negative mediating cytokine, plays a pivotal role in the maintenance of immune homeostasis. Specifically, the feature function of T cells and APCs is limited by it. This interference serves to safeguard normal tissue from the detrimental effects of inflammation.^[50]^ LAG-3 deficiency in T cells has been found to play a significant role in their unrestrained proliferation and immune function.^[51]^ Additionally, CD4^+^T cells have been shown to drive humoral immunity, leading to the production of self-antibodies.^[52,53]^ Regarding Tregs, the expression of LAG-3 is stimulated by IL-27, which subsequently binds to the surface MHCII of naïve DCs to impede their maturation process.^[54–56]^ The LAG-3 molecule has the ability to downregulate the antigen presenting effectiveness of APCs by inhibiting the glycolytic pathway.^[21]^ In prevalent autoimmune disorders such as inflammatory bowel disease and rheumatoid arthritis, the dysregulation of LAG-3 plays a significant role in their pathogenesis.^[30,57]^

Specifically, LAG-3 deficiency does not directly induce autoimmune symptoms in nonautoimmune susceptible mice. However, it does accelerate the development of related diseases, as evidenced in the case of type I diabetes (T1D).^[58]^ A specific type of T cell found in islets, exhibit similarities in phenotype characteristics to exhausted T cell. However, there are still some distinguishing characteristics, and these T cells are referred to as “restrained” T cells.^[59]^ In a mouse model of T1D, disease progression was accelerated following the removal of LAG-3 from the surface of CD8+ T cells, and the “restrained” phenotype was also alleviated. Constant depletion of LAG-3 on the surface of Tregs prevents the development of T1D in mice. These phenomena suggest that both CD4+ and CD8+ T cells play a role in accelerating the development of T1D, challenging the traditional belief that only CD4+ T cells are involved. This effect overrides the function of Treg cells in preventing disease formation. The elucidation of the mechanism by which LAG-3 elicits the “restraint” of CD8+ T cell requires additional investigation.^[59]^

In the context of autoimmune disease, it is critical to consider the role of LAG-3 in maintaining immune homeostasis. To restore immune tolerance or improve symptoms, the upregulation of LAG-3 expression levels and activation of its signaling pathway are potential medical interventions. IMP-761, an agonistic antibody, effectively meets the aforementioned requirements and exhibits specific binding to LAG-3+ T cells. Consequently, this leads to the inhibition of T cell proliferation and immune function.^[60]^ Additionally, as previously mentioned, soluble FGL-1 serves as the primary ligand that facilitates the utilization of LAG-3’s immunosuppressive function. Based on that, researchers discovered that additional soluble FGL-1 effectively mitigated the inflammatory phenotype in a mouse model of arthritis. Importantly, this treatment did not result in any immune-related adverse effects in other organs.^[61]^ Furthermore, LAG-3 has been identified as a prognostic biomarker for certain autoimmune diseases.^[62–64]^ In the case of patients with rheumatoid arthritis, the presence of peripheral sLAG-3 is primarily derived from the cleaved surface LAG-3 of Tregs. Thus, the level of peripheral sLAG-3 is positively correlated with the severity of the illness, as it is a favorable prognosis biomarker in patients with MS.^[63]^

### 5.2. Antitumor immunity

If an excessive deficiency of LAG-3 leads to the manifestation of various autoimmune phenomena, then an excessive expression of LAG-3 results in tumor immune evasion. The fundamental cause, as previously mentioned, is the excessive presence of LAG-3, which restricts T cell functionality and leads to a state of “exhaustion.” The origin of LAG-3 can be attributed to persistent stimulation, which is derived from the solid tumor.^[65,66]^ Hence, the reversal of exhaustion is regarded as the primary approach for immune therapy, and reducing the functioning of LAG-3 is the specific method.^[4,67]^

As a novel therapeutic target following PD-1 and cytotoxic T lymphocyte-associated antigen-4, LAG-3 exhibits functional similarities to PD-1, as well as shared limitations. Solely targeting either PD-1, LAG-3, cytotoxic T lymphocyte-associated antigen-4, or TIM-3 with mAbs can lead to the upregulation of the expression of other ICs. This compensatory effect facilitates tumor resistance to McAbs.^[68]^ To mitigate this phenomenon, the implementation of dual- or tri- combination immunotherapy arises as a more precise and efficacious strategy for treatment.^[69,70]^ In a research study, scientists developed mouse models that were resistant to anti-LAG-3 or anti-PD-1 treatment. The utilization of a combination therapy involving dual antibodies demonstrated an improved efficacy. Additionally, no immune-related adverse effects were observed.^[71]^ In several tumor models, specifically advanced melanoma, the simultaneous inhibition of LAG-3 and PD-1 demonstrates a more significant antitumor effect when compared to the use of individual McAbs.^[71–74]^ The combination of Nivolumab with Relatlimab has shown significant advantages compared to Nivolumab monotherapy in a phase II/III clinical trial.^[75]^

Is it possible to combine 2 or 3 ICIs into a single molecule? The hypothesis was fulfilled by the bispecific antibody (bsAb). These bsAbs have already been created and are currently undergoing clinical trials. Compared to traditional single or dual McAb therapy, bsAbs offer functional advantages. First, a bsAb allows for the simultaneous targeting of 2 specific antigens, thereby enhancing the precision of drug effects. This dual targeting capability also helps to minimize harm to normal cells, reducing potential side effects. Second, assist lymphocytes in locating and disrupting tumor cells. Third, 2 tumor cell signals were simultaneously inhibited.^[76]^ Various mechanisms contribute to the enhanced accuracy and efficiency of tumor growth and invasion inhibition by bsAbs. Tebotelima aims to inhibit the simultaneous interaction between PD-1/LAG-3 and their ligands in order to restore the functionality of exhausted T cells. Phase II/III MAHOGANY experiments have also demonstrated the efficacy of Tebotelima in restricting tumor growth.^[77]^ FS118, when used in combination to block LAG-3 and PD-L1, primarily enhances the antigen cross-presentation function of DCs by promoting their maturation. Consequently, this combination activates more CD8+ T cells and suppresses tumor growth in vivo.^[78]^ More extensive clinical trials are necessary to gain a deeper understanding of the precise functions of these drugs and to evaluate the synergistic effects of different therapies. This will provide more reliable references for their widespread application.

In terms of prognosis, LAG-3 is also a biomarker used to assess the effectiveness of immune therapy. As mentioned before, the increase in LAG-3 is indicative of tumor drug resistance to any single mAb. Therefore, it can be recognized as an independent biomarker for poor prognosis.^[79–83]^ However, relying solely on LAG-3 is insufficient for evaluating tumor conditions. Various TILs also participate in this process, not to mention the variations among individuals. Combining the surveillance on ICs and TILs is required to provide personalized and accurate clinical treatment. In triple negative breast cancer, studies have shown that LAG-3+ CD8+ cells can serve as a protective and favorable prognostic biomarker.^[84,85]^ Another study^[67]^ found that mAb or bsAb effectively reversed T cell exhaustion. This was further confirmed by observing increased apoptosis in tumor cells and enhanced proliferation of lymphocytes. These findings suggest that targeting CD8+ TILs is a main pathway for effective targeted therapy. Therefore, monitoring LAG-3+ CD8+ cells could provide valuable insights for developing individualized targeted therapy strategies.

## 6. Conclusion

LAG-3, a crucial regulator of immune homeostasis, exerts a significant influence on both autoimmune and antitumor immune responses. In this review, we provide an overview of the research conducted in recent years on LAG-3, covering its fundamental aspects, clinical applications, and its role in both autoimmunity and antitumor immunity. Several significant findings have emerged regarding the potential of LAG-3 targeted drugs to enhance their widespread application. However, there are still unresolved issues that need to be addressed. The downstream signal transduction pathways of LAG-3, the influence of LAG-3 on non-T lymphocytes, and the cooperative mechanisms of combined ICI therapies targeting LAG-3 and PD-1 in various TMEs. Further experimentation is necessary to ensure the safe and widespread implementation of new strategies for treating cancer and autoimmune disorders.

## Author contributions

**Investigation:** Xue Qin.

**Writing – original draft:** Jiaqi Nie.

**Writing – review & editing:** Xiang Tao, Jin Huang.
